# Brief Report: Safety and Antitumor Activity of Alectinib Plus Atezolizumab From a Phase 1b Study in Advanced *ALK*-Positive NSCLC

**DOI:** 10.1016/j.jtocrr.2022.100367

**Published:** 2022-06-25

**Authors:** Dong-Wan Kim, Shirish Gadgeel, Scott N. Gettinger, Gregory J. Riely, Geoffrey R. Oxnard, Tarek Mekhail, Peter Schmid, Afshin Dowlati, Rebecca S. Heist, Antoinette J. Wozniak, Jatinder Singh, Edward Cha, Jessica Spahn, Sai-Hong Ignatius Ou

**Affiliations:** a Department of Internal Medicine, Seoul National University College of Medicine and Seoul National University Hospital, Seoul, South Korea; b Department of Internal Medicine, Henry Ford Cancer Institute, Henry Ford Health System, Detroit, Michigan; c Department of Medicine (Medical Oncology), Yale School of Medicine, New Haven, Connecticut; d Division of Solid Tumor Oncology, Department of Medicine, Memorial Sloan Kettering Cancer Center, New York, New York; e Dana-Farber Cancer Institute and Harvard Medical School, Boston, Massachusetts; f AdventHealth Cancer Institute, Orlando, Florida; g Centre for Experimental Cancer Medicine, Barts Cancer Institute, Queen Mary University of London, London, United Kingdom; h Division of Hematology and Oncology, University Hospitals Seidman Cancer Center and Case Western Reserve University, Cleveland, Ohio; i Massachusetts General Hospital Cancer Center, Boston, Massachusetts; j UPMC Hillman Cancer Center, University of Pittsburgh, Pittsburgh, Pennsylvania; k Genentech, Inc., South San Francisco, California; l University of California Irvine School of Medicine, Orange, California

**Keywords:** Alectinib, Atezolizumab, *ALK*-positive, Non–small cell lung cancer, Phase 1b study

## Abstract

**Introduction:**

Alectinib is a preferred first-line treatment option for advanced *ALK*-positive NSCLC. Combination regimens of alectinib with immune checkpoint inhibitors are being evaluated for synergistic effects.

**Methods:**

Adults with treatment-naive, stage IIIB/IV, or recurrent *ALK*-positive NSCLC were enrolled into a two-stage phase 1b study. Patients received alectinib 600 mg (twice daily during cycle 1 and throughout each 21-d cycle thereafter) plus atezolizumab 1200 mg (d8 of cycle 1 and then d1 of each 21-d cycle). Primary objectives were to evaluate safety and tolerability of alectinib plus atezolizumab. Secondary objectives included assessments of antitumor activity.

**Results:**

In total, 21 patients received more than or equal to 1 dose of alectinib or atezolizumab. As no dose-limiting toxicities were observed in stage 1 (n = 7), the starting dose and schedule were continued into stage 2 (n = 14). Median duration of follow-up was 29 months (range: 1–39). Grade 3 treatment-related adverse events occurred in 57% of the patients, most often rash (19%). No grade 4 or 5 treatment-related adverse events were reported. Confirmed objective response rate was 86% (18 of 21; 95% confidence interval [CI]: 64–97). Median progression-free survival was not estimable (NE) (95% CI: 13 mo–NE), neither was median overall survival (95% CI: 33 mo–NE).

**Conclusions:**

The combination of alectinib and atezolizumab is feasible, but increased toxicity was found compared with the individual agents. With small sample sizes and relatively short follow-up, definitive conclusions regarding antitumor activity cannot be made.

## Introduction

*ALK* gene rearrangements (*ALK*-positive) are oncogenic drivers in approximately 5% of patients with advanced NSCLC.^1^ Alectinib is a next-generation *ALK* tyrosine kinase inhibitor (TKI) that is approved and recommended as a preferred first-line treatment option for advanced *ALK*-positive NSCLC.^2^ In the global phase 3 ALEX study, alectinib significantly improved progression-free survival (PFS) compared with crizotinib (median PFS = 34.8 versus 10.9 mo, stratified hazard ratio [HR] = 0.43, 95% confidence interval [CI]: 0.32–0.58) and produced a clinically meaningful improvement in 5-year overall survival (OS) in patients with treatment-naive *ALK*-positive NSCLC.^3^

Atezolizumab is a humanized monoclonal antibody that blocks the interaction between programmed death-ligand 1 (PD-L1) and programmed cell death protein 1 and B7.1 (CD80).^4^ Atezolizumab significantly improved OS versus docetaxel in previously treated NSCLC in the randomized, phase 3 OAK study (median OS = 13.8 versus 9.6 mo; HR = 0.73; 95% CI: 0.62–0.87).^4^ Significantly prolonged OS was also reported with atezolizumab relative to platinum-based chemotherapy in the randomized, phase 3 IMpower110 study in patients with NSCLC and high PD-L1 expression (median OS = 20.2 versus 13.1 mo; stratified HR = 0.59; 95% CI: 0.40–0.89).^5^ Atezolizumab is approved as monotherapy or in combination with chemotherapy, with or without bevacizumab, for several NSCLC indications.^6^ Approval of the first-line combination regimen of atezolizumab plus bevacizumab and chemotherapy was based on results of the phase 3 IMpower150 study. The combination regimen produced longer OS and PFS than bevacizumab plus chemotherapy alone in the intent-to-treat population^7^^,^^8^; in addition, prolonged PFS was reported in the subgroup of patients with *EGFR*-mutant or *ALK-*positive NSCLC (median PFS = 9.7 versus 6.1 mo, HR = 0.59, 95% CI: 0.37–0.94).^7^ No OS benefit was observed in the intent-to-treat population with the combination of atezolizumab plus chemotherapy versus bevacizumab plus chemotherapy.^8^

Increased tumor cell death is associated with more effective release and presentation of tumor antigens, which broadens the T-cell response. Thus, combining alectinib, a TKI that leads to antigen release, with atezolizumab, an immune checkpoint inhibitor (ICI) that releases T-cell inhibition to drive tumor cell death, has the potential to produce additive and durable antitumor effects in patients with *ALK*-positive NSCLC. Nevertheless, limited data are available. Here, we report the safety, tolerability, and preliminary antitumor activity of alectinib in combination with atezolizumab in patients with advanced *ALK*-positive NSCLC.

## Materials and Methods

This phase 1b, open-label, multicenter study investigated the combination of alectinib plus atezolizumab in patients with advanced *ALK*-positive NSCLC and erlotinib plus atezolizumab in patients with advanced *EGFR*-mutant NSCLC (NCT02013219). It was conducted in the following two stages: safety evaluation (stage 1) and expansion (stage 2). We report results from the alectinib plus atezolizumab arm only.

During stage 1, patients received alectinib 600 mg orally twice daily during cycle 1 (28d) and throughout each 21-day cycle thereafter. Atezolizumab 1200 mg was administered intravenously on day 8 of cycle 1 and on day 1 of each subsequent 21-day cycle. In stage 2, the potential recommended phase 2 dose and schedule were investigated in an expansion cohort on the basis of the maximum tolerated dose identified in stage 1. Additional details regarding the methodology are included in the Supplementary Materials.

Eligible patients were aged more than or equal to 18 years with treatment-naive, measurable (by Response Evaluation Criteria in Solid Tumors version 1.1), histologically or cytologically confirmed, stage IIIB/IV or recurrent NSCLC. Other key inclusion criteria included the following: confirmed *ALK*-positive status, adequate hematologic and end-organ function, Eastern Cooperative Oncology Group performance status score of 0 or 1, and life expectancy of more than or equal to 12 weeks. Patients were excluded if they had the following: received an approved anticancer therapy within 3 weeks before the initiation of study treatment; received prior CD137 agonists, ICIs, systemic immunostimulatory agents, or systemic immunosuppressive medications; or had known primary central nervous system (CNS) malignancy or symptomatic CNS metastases.

The primary objective of stage 1 was to identify any dose-limiting toxicities (DLTs). Additional primary objectives (for stages 1 and 2) were safety and tolerability evaluations of alectinib plus atezolizumab and to identify the recommended phase 2 dose and schedule. Secondary objectives were to make a preliminary assessment of the antitumor activity of alectinib plus atezolizumab and to characterize the pharmacokinetics and immunogenic potential of the combination therapy.

This study was approved by local institutional review boards or ethics committees. Written informed consent was obtained from all patients, in accordance with the principles of the Declaration of Helsinki.

## Results

### Patients

A total of 22 patients were enrolled between September 9, 2015, and March 31, 2017, across sites in South Korea, Spain, United Kingdom, and United States. At data cutoff (May 7, 2020), 21 patients who had received more than or equal to 1 dose of alectinib or atezolizumab were considered assessible for response and safety (study stage 1, n = 7; study stage 2, n = 14); one patient discontinued before treatment administration owing to elevated alanine aminotransferase (ALT) level. All 21 patients had discontinued study treatments (Supplementary Table 1). Baseline demographics and disease characteristics are summarized in Table 1.

**Table 1:** Baseline Demographics and Disease Characteristics (Safety-Evaluable Patients)

Characteristics	Category	All Patients (N = 21)
Age, y	Median (range)	53 (36‒75)
Sex, n (%)	Female	9 (43)
	Male	12 (57)
Race, n (%)	Asian	9 (43)
	White	12 (57)
Smoking status, n (%)	Current	2 (10)
	Prior	6 (29)
	Never	13 (62)
Histology type, n (%)	Squamous	2 (10)
	Non-squamous	19 (90)
ECOG PS, n (%)	0	8 (38)
	1	13 (62)
CNS metastasis, n (%)	Present	6 (29)
	Absent	15 (71)
Prior systemic anticancer therapy, n (%)	Yes^a^	2 (10)
	No	19 (90)
Prior radiotherapy, n (%)	Yes	1 (5)
	No	20 (95)

CNS, central nervous system; ECOG PS, Eastern Cooperative Oncology Group performance status.

a Cisplatin, vinorelbine, carboplatin, pemetrexed, and crizotinib (n = 1) and carboplatin and paclitaxel (n = 1) before starting the study.

### Safety

The median duration of follow-up was 29 months (range: 1–39). With alectinib, the median duration of treatment was 22 months (1–39) and the median number of doses per cycle was 34 (range: 1–56). The median duration of treatment with atezolizumab was 10 months (range: 0–38), and the median number of doses was 15 (range: 1–56).

As no DLTs were reported in stage 1, the starting dose and schedule were continued into stage 2. The most frequently reported treatment-emergent adverse events (AEs) are presented in Supplementary Table 2. Grade 3 treatment-related AEs were experienced by 57% of patients, with rash (19%) being the most frequently reported (Table 2). There were no grade 4 or 5 treatment-related AEs. Alectinib-related AEs were observed in 90% of the patients and atezolizumab-related AEs in 86% of the patients (Table 2). AEs of special interest related to liver and lung function occurred at rates below 30% (Supplementary Table 3); the most often occurring grade 3 events were rash (19%), increased blood bilirubin level (10%), and increased ALT level (10%). Of these, 33.3% of the patients required treatment with systemic corticosteroids.

**Table 2:** Treatment-Related AEs (Safety-Evaluable Population)

Preferred Term, n (%)	Treatment-Related AEs		Alectinib-Related AEs		Atezolizumab-Related AEs	
	Any Grade	Grade 3^a^	Any Grade	Grade 3^a^	Any Grade	Grade 3^a^
**Any AE**	**20 (95)**	**12 (57)**	**19 (90)**	**10 (48)**	**18 (86)**	**9 (43)**
Rash	11 (52)	4 (19)	6 (29)	2 (10)	10 (48)	4 (19)
Blood bilirubin increased	6 (29)	2 (10)	6 (29)	2 (10)	1 (5)	−
ALT increased	5 (24)	2 (10)	5 (24)	2 (10)	5 (24)	2 (10)
Dyspnea	2 (10)	2 (10)	1 (5)	1 (5)	2 (10)	2 (10)
Liver function test increased^b^	2 (10)	1 (5)	1 (5)	1 (5)	2 (10)	1 (5)
Neutropenia	1 (5)	1 (5)	1 (5)	1 (5)	1 (5)	1 (5)
Hypophosphatemia	1 (5)	1 (5)	1 (5)	1 (5)	1 (5)	−
Aseptic meningitis	1 (5)	1 (5)	−	−	1 (5)	1 (5)
Pneumonitis	1 (5)	1 (5)	−	−	1 (5)	1 (5)

AE, adverse event; ALT, alanine aminotransferase.

a No grade 4 or 5 treatment-related AEs were reported.

b Term used by investigators if multiple liver enzyme tests were elevated in a single patient.

AEs resulted in discontinuation of atezolizumab in seven patients (33%) and to discontinuation of alectinib in four patients (19%; Supplementary Table 4). A total of 27 AEs resulted in alectinib dose modification or interruption in 14 patients (67%), most often increased ALT level, dyspnea, and increased blood creatine phosphokinase level (10% each). Overall, 19 AEs led to the interruption of atezolizumab treatment in eight patients (38%), most often diarrhea (14%).

### Antitumor Activity

Investigator-assessed confirmed objective response rate (ORR) was 86% (18 of 21, 95% CI: 64–97; Table 3). Median duration of response was not estimable (NE, 95% CI: 12 mo–NE; Supplementary Fig. 1). Most responses were observed by 4 months and were sustained over time (Supplementary Fig. 2). Of the patients with CNS metastases at baseline, five (83%) experienced a partial response and one (17%) was not evaluable. Median PFS was not evaluable (13 mo–NE; Table 3 and Supplementary Fig. 3*A*) and eight patients (38%) experienced a PFS event (n = 7 progressive disease; n = 1 death). Median OS was not evaluable (33 mo–NE; Table 3 and Supplementary Fig. 3*B*); five patients (24%) had died at the time of data cutoff.

**Table 3:** Antitumor Activity (Efficacy-Evaluable Population)

Outcomes	All Patients (N = 21)
Progression-free survival	
Median, mo (95% CI)	NE (13‒NE)
6-mo rate, % (95% CI)	95 (85‒100)
12-mo rate, % (95% CI)	72 (52‒93)
24-mo rate, % (95% CI)	56 (33‒79)
Overall survival	
Median, mo (95% CI)	NE (33‒NE)
6-mo rate, % (95% CI)	100 (100‒100)
12-mo rate, % (95% CI)	94 (84‒100)
24-mo rate, % (95% CI)	78 (59‒97)
Confirmed objective response rate, n (%; 95% CI)	18 (86; 64‒97)
Complete response	3 (14; 3‒36)
Partial response	15 (71; 48‒89)
Stable disease	2 (10; 1‒30)
Missing/unevaluable, n (%)	1 (5)
Disease control rate, n (%; 95% CI)	19 (90; 70‒99)
Duration of response	
Median, mo (95% CI)	NE (12‒NE)
6-mo rate, % (95% CI)	88 (73‒100)
12-mo rate, % (95% CI)	71 (49‒92)

CI, confidence interval; NE, not estimable.

### Biomarkers

Among patients with PD-L1–evaluable tumors, PD-L1 expression (immune cell [IC]0 and tumor cell [TC]0 [<1%]; IC1 or TC1 [≥1% but <5%]; TC2 [≥5% but <50%]) was observed in eight of 15 patients (53.3%) at baseline (Supplementary Fig. 4). Two patients had PD-L1 expression on both immune and tumor cells, and six patients had PD-L1 expression on tumor cells only. CD8 T-cell counts were evaluable in 17 of 20 patients at baseline and ranged from 0.2% to 15% of the central tumor area, with a median of 0.8%. CD8 T-cell count increases were observed post-alectinib run-in in seven of nine paired biopsies collected at screening and day 7 of cycle 1 (Supplementary Fig. 5); however, no clear association with response was found with CD8 detection.

## Discussion

First-line treatment with next-generation TKIs has improved outcomes for patients with advanced *ALK*-positive NSCLC; however, most patients eventually relapse. It was hypothesized that alectinib plus atezolizumab may provide additive or more prolonged clinical benefit in this setting, prompting this evaluation of the toxicity and feasibility of the combination.

The nature of reported AEs was consistent with both alectinib and atezolizumab monotherapy, but the combination regimen led to an increased rate of AEs and dose discontinuations or modifications compared with the individual agents.^3^, ^4^, ^5^, ^6^^,^^9^, ^10^, ^11^ The rate of any-grade AEs reported by patients receiving alectinib plus atezolizumab in this study was similar to patients who were treated with alectinib monotherapy in the ALEX study (97%)^9^ and those who received atezolizumab monotherapy in the phase 3 OAK (94%) and IMpower110 studies (90%).^4^^,^^5^ Nevertheless, a higher rate of grade more than or equal to 3 AEs was found with alectinib plus atezolizumab combination therapy in this study compared with single-agent alectinib or atezolizumab (67% versus 30%–41%, respectively).^4^^,^^5^^,^^9^

All combination regimens of ALK TKIs with ICIs have reported increased toxicity compared with the individual agents alone. Severe hepatotoxicity was found in 38% of patients when treated with crizotinib plus nivolumab, leading to early study termination.^10^ Similarly, excessive short-term gastrointestinal toxicity led to the early discontinuation of a study evaluating the combination of crizotinib and ipilimumab.^11^ A high rate of DLTs was found in a phase 1 study evaluating the combination of crizotinib plus pembrolizumab,^12^ although the study was terminated early owing to the slow enrolment of patients. Recently, an increase in toxicity, including incidence of rash, was found with the combination of ceritinib plus nivolumab in comparison with the individual agents.^13^ In contrast, the combination of lorlatinib plus avelumab was found to have an acceptable safety profile in patients who have previously received treatment (a median of 2 prior ALK TKIs).^14^

The antitumor activity of alectinib plus atezolizumab was consistent with that previously reported in patients with advanced *ALK*-positive NSCLC treated with alectinib monotherapy for a similar duration in ALEX.^9^ Here, we report a confirmed ORR of 86% (95% CI: 64–97), which is numerically higher than the confirmed ORR reported in the primary analysis of the ALEX study (71.7% [95% CI: 63.8–78.7]).^15^ The PFS rates were similar between those of the present analysis (72% [95% CI: 52–93]) and ALEX (68.4% [95% CI: 61.0–75.9]) at 12 months and at 24 months (present analysis: 56% [95% CI: 33–79] and ALEX: 56.6%).^3^^,^^9^

Although no unexpected safety findings were identified with the combination of alectinib and atezolizumab, the incidence of treatment-related grade 3 AEs and treatment discontinuations of both drugs owing to AEs were higher than those reported with either drug alone in prior clinical trials. The antitumor activity of the combination was broadly similar to alectinib monotherapy. Nevertheless, given the relatively short follow-up, definitive conclusions regarding antitumor activity cannot be made. In light of the differences in study design and the very small sample size in the present study, any comparisons with single-agent and other combination studies should be interpreted with caution.

## CRediT Authorship Contribution Statement

**Dong-Wan Kim:** Investigation, resources, writing—review and editing.

**Shirish Gadgeel**: Investigation, resources, writing—review and editing.

**Scott N. Gettinger:** Conceptualization, investigation, resources, writing—review and editing, supervision, project administration.

**Gregory J. Riely:** Investigation, resources, writing—review and editing, funding acquisition.

**Geoffrey R. Oxnard:** Investigation, writing—review and editing.

**Tarek Mekhail**: Investigation, data curation, writing—review and editing.

**Peter Schmid:** Formal analysis, investigation, writing—original draft, writing—review and editing, supervision.

**Afshin Dowlati:** Formal analysis, investigation, resources, data curation, writing—review and editing, supervision.

**Rebecca S. Heist:** Investigation, writing—review and editing.

**Antoinette J. Wozniak:** Investigation, writing—review and editing.

**Jatinder Singh:** Formal analysis, writing—review and editing.

**Edward Cha:** Conceptualization, methodology, formal analysis, writing—review and editing, supervision.

**Jessica Spahn**: Methodology, writing—original draft, writing—review and editing, visualization, supervision.

**Sai-Hong Ignatius Ou:** Methodology, investigation, resources, writing—review and editing, visualization.

## Data Sharing Statement

The study is not in scope according to the Roche global policy on data sharing because it is a phase 1b study. The decision to share the patient-level data needs to be handled on a case-by-case basis to determine whether the data can be adequately anonymized to give an acceptably low risk of patient reidentification. Qualified researchers may submit an enquiry through the data request platform, Vivli, https://vivli.org/ourmember/roche/; however, this does not guarantee that the data can be shared. For up-to-date details on Roche’s Global Policy on the Sharing of Clinical Information and how to request access to related clinical study documents, see here: https://www.roche.com/innovation/process/clinical-trials/data-sharing/. Anonymized records for individual patients across more than one data source external to Roche cannot, and should not, be linked owing to a potential increase in risk of patient reidentification.

## References

[1] Barlesi F., Mazieres J., Merlio J.P. (2016). Routine molecular profiling of patients with advanced non-small-cell lung cancer: results of a 1-year nationwide programme of the French Cooperative Thoracic Intergroup (IFCT). Lancet.

[2] Hanna N.H., Robinson A.G., Temin S. (2021). Therapy for stage IV non-small-cell lung cancer with driver alterations: ASCO and OH (CCO) joint guideline update. J Clin Oncol.

[3] Mok T., Camidge D.R., Gadgeel S.M. (2020). Updated overall survival and final progression-free survival data for patients with treatment-naive advanced *ALK*-positive non-small-cell lung cancer in the ALEX study. Ann Oncol.

[4] Rittmeyer A., Barlesi F., Waterkamp D. (2017). Atezolizumab versus docetaxel in patients with previously treated non-small-cell lung cancer (OAK): a phase 3, open-label, multicentre randomised controlled trial. Lancet.

[5] Herbst R.S., Giaccone G., de Marinis F. (2020). Atezolizumab for first-line treatment of PD-L1-selected patients with NSCLC. N Engl J Med.

[6] Food & Drug Administration (FDA) Atezolizumab Highlights of Prescribing information. https://www.accessdata.fda.gov/drugsatfda_docs/label/2020/761034s027lbl.pdf.

[7] Socinski M.A., Jotte R.M., Cappuzzo F. (2018). Atezolizumab for first-line treatment of metastatic nonsquamous NSCLC. N Engl J Med.

[8] Reck M., Mok T.S.K., Nishio M. (2019). Atezolizumab plus bevacizumab and chemotherapy in non-small-cell lung cancer (IMpower150): key subgroup analyses of patients with EGFR mutations or baseline liver metastases in a randomised, open-label phase 3 trial. Lancet Respir Med.

[9] Peters S., Camidge D.R., Shaw A.T. (2017). Alectinib versus crizotinib in untreated *ALK*-positive non-small-cell lung cancer. N Engl J Med.

[10] Spigel D.R., Reynolds C., Waterhouse D. (2018). Phase 1/2 Study of the safety and tolerability of nivolumab plus crizotinib for the first-line treatment of anaplastic lymphoma kinase translocation-positive advanced non-small cell lung cancer (CheckMate 370). J Thorac Oncol.

[11] Chalmers A.W., Patel S., Boucher K. (2019). Phase I trial of targeted EGFR or ALK therapy with ipilimumab in metastatic NSCLC with long-term follow-up. Target Oncol.

[12] Patel S.P., Pakkala S., Pennell N.A. (2020). Phase Ib study of crizotinib plus pembrolizumab in patients with previously untreated advanced non-small cell lung cancer with *ALK* translocation. Oncologist.

[13] Felip E., de Braud F.G., Maur M. (2020). Ceritinib plus nivolumab in patients with advanced ALK-rearranged non-small cell lung cancer: results of an open-label, multicenter, phase 1B study. J Thorac Oncol.

[14] Shaw A.T., Lee S.-H., Ramalingam S.S. (2018). Avelumab (anti–PD-L1) in combination with crizotinib or lorlatinib in patients with previously treated advanced NSCLC: phase 1b results from JAVELIN Lung 101. J Clin Oncol.

[15] Mok T., Shaw A.T., Camidge D.R. (2019). 1484-PD Final PFS, updated OS and safety data from the randomised, phase III ALEX study of alectinib versus crizotinib in untreated advanced *ALK* + NSCLC (Poster presented at ESMO 2019). Ann Oncol.

