# Supplementary material for: Brief Report: Safety and Antitumor Activity of Alectinib Plus Atezolizumab From a Phase 1b Study in Advanced ALK-Positive NSCLC
Source: JTO Clin Res Rep. 2022 Jun 25;3(8):100367. doi: 10.1016/j.jtocrr.2022.100367 (PMC9304608; doi:10.1016/j.jtocrr.2022.100367)
Supplement: Supplementary Material [file mmc1.docx]

**Supplementary Material**

**Brief Report: Safety and Anti-Tumor Activity of Alectinib Plus Atezolizumab from a Phase 1b Study in Advanced *ALK*-Positive NSCLC**

Dong-Wan Kim et al.

**Materials and Methods**

**Exclusion criteria**

Patients were excluded if they had received an approved anti-cancer therapy within 3 weeks prior to the initiation of study treatment, including chemotherapy or hormonal therapy (excluding hormone-replacement therapy or oral contraceptives), or if they had a known primary central nervous system (CNS) malignancy or symptomatic CNS metastases (patients with asymptomatic treated or untreated CNS disease may be enrolled according to defined criteria), leptomeningeal disease, symptomatic bradycardia, or a QT interval >470 ms. Patients who had received prior CD137 agonists, immune checkpoint inhibitors, systemic immunostimulatory agents, or systemic immunosuppressive medications were also not eligible for enrollment.

**Dosage modification protocol**

In the event of a dose-limiting toxicity (DLT) or other adverse event (AE) of concern, a maximum of two 150 mg dose reductions of alectinib were permitted. If alectinib 300 mg twice daily was still not tolerated, treatment with alectinib was discontinued; however, treatment with atezolizumab could continue at the investigator’s discretion. Alectinib could be interrupted for up to 21 days due to treatment-related toxicity that could not be controlled by supportive medication. Atezolizumab dose modification was not permitted but could be interrupted in response to atezolizumab-related AEs. If the starting regimen was not tolerated, alternative doses/schedules may be tested to define a potential recommended phase 2 dose for combination treatment. Treatment of alectinib and atezolizumab combination therapy continued beyond cycle 1 at the discretion of the investigator, until there was evidence of progressive disease by Response Evaluation Criteria in Solid Tumors [RECIST v1.1]), unacceptable toxicity, or lack of clinical benefit.

**Dose-limiting toxicities**

DLTs were defined as:

- Grade ≥4 thrombocytopenia lasting ≥7 days or thrombocytopenia associated with bleeding
- Grade ≥4 neutropenia lasting ≥7 days
- Grade ≥3 symptomatic hepatic toxicities lasting for >48 hours, or grade ≥3 asymptomatic hepatic toxicities lasting for >7 days with the following exceptions:
  - For patients with grade 2 alkaline phosphatase abnormality at baseline, an increase to more than eight times the upper limit of normal lasting >48 hours (symptomatic) or >7 days (asymptomatic)
  - For asymptomatic patients with grade 2 aspartate aminotransferase (AST) and/or alanine aminotransferase (ALT) at baseline, grade 3 AST or ALT elevation for 7 days or grade 4 AST or ALT
- Grade ≥3 non-hematologic, non-hepatic organ toxicity, excluding the following:
  - Grade 3 nausea, vomiting or diarrhea that resolves to grade ≤1 within 7 days of appropriate supportive therapy
  - Grade ≥3 asymptomatic or mildly symptomatic rash that can be adequately managed with supportive care or resolves to become asymptomatic and/or Grade ≤2 within 7 days of appropriate supportive therapy
  - Grade ≥3 fatigue that resolves to grade ≤2 within 7 days
  - Grade ≥3 asymptomatic elevation of serum creatine phosphokinase (CPK) level that is deemed to be clinically insignificant by the investigator that returns to grade ≤2 within 7 days of alectinib treatment interruption
  - Grade 3 arthralgia that can be adequately managed with supportive care or that resolves to grade ≤2 within 7 days

**Assessments**

Anaplastic lymphoma kinase (*ALK)*-positive status was confirmed by local *ALK* testing using a US Food and Drug Administration (FDA)-approved assay. AEs were graded using National Cancer Institute Common Terminology Criteria for Adverse Events v4.0; AEs of special interest related to atezolizumab were also evaluated. Anti-tumor activity outcome measures included progression free survival, confirmed objective response rate, duration of response, as assessed by investigators using RECIST v1.1, and overall survival. Objective response rate was defined as a complete response or partial response and confirmed by repeat assessments ≥4 weeks after initial documentation. Blood samples were collected at Cycle 1 Day 1, 8 and 15, and Day 1 of every cycle thereafter for PK and biomarker assessment. A formalin-fixed, paraffin-embedded tumor specimen collected at first diagnosis or subsequent tumor recurrence was required for all patients. Tissue biopsies were collected at baseline, Cycle 1 Day 7, 4–6 weeks after the first atezolizumab dose, and at PD; on-treatment and PD biopsies were optional in stage 1 and mandatory in stage 2. PD-L1 expression and CD8+ T‑cell count were determined by immunohistochemistry (VENTANA PD-L1 [SP142] and HistoGeneX [C8/144B; DAKO]), using archival and/or newly collected tissue samples. CD8 levels were reported as percentages and refer to the area within the tumor central region with CD8 staining.

**Statistical analysis**

No formal hypothesis testing was planned. Sample size was based on dose-modification rules and the probability of observing AEs or DLTs by pre-defined precision estimates on AE incidence. The study planned to enroll approximately 6–12 patients in stage 1 and 14 patients in stage 2; the sample size could be increased if patients in stage 1 were found to be unevaluable for DLTs. Safety assessments were conducted in the safety-evaluable population, which included all patients who received any dose of study treatment. Preliminary anti-tumor activity was determined in the efficacy-evaluable population, which included patients who received at least one dose of atezolizumab and alectinib and had measurable disease at baseline (RECIST v1.1).

**SUPPLEMENTARY TABLES**

**Supplementary Table 1.** Reasons for Study and Treatment Discontinuation (Safety-Evaluable Population)

| **Primary Reason for Discontinuation, n (%)** | **Study** | **Treatment** | |
| --- | --- | --- | --- |
|  |  | **Alectinib** | **Atezolizumab** |
| Study termination | 12 (57) | 9 (43) | 7 (33) |
| AE | 2 (10) | 4 (19) | 6 (29) |
| PD | 1 (5) | 7 (33) | 6 (29) |
| Death | 5 (24)^a^ | 1 (5) | 1 (5) |
| Withdrawal of consent | 1 (5) | 0 | 0 |
| Other | 0 | 0 | 1 (5) |

^a^The study discontinuations due to death were a result of PD (n = 4) and due to unknown causes (n = 1).

AE, adverse event; PD, progressive disease.

**Supplementary Table 2.** Treatment-Emergent Adverse Events Occurring in ≥20% of Patients (Safety-Evaluable Population)

| **Preferred term, n (%)** | **Any Grade** | **Grade 3^a^** |
| --- | --- | --- |
| **Any AE** | **21 (100)** | **14 (67)** |
| Rash | 12 (57) | 4 (19) |
| Constipation | 11 (52) | − |
| Fatigue | 10 (48) | − |
| Pyrexia | 9 (43) | 2 (10) |
| Cough | 8 (38) | − |
| Myalgia | 7 (33) | − |
| Dizziness | 7 (33) | − |
| Headache | 7 (33) | − |
| Abdominal pain | 6 (29) | − |
| AST increased | 6 (29) | − |
| Blood bilirubin increased | 6 (29) | 2 (10) |
| Dyspnea | 6 (29) | 2 (10) |
| ALT increased | 5 (24) | 2 (10) |
| Pneumonia | 5 (24) | 2 (10) |
| Diarrhea | 5 (24) | 1 (5) |
| Nausea | 5 (24) | − |
| Pruritus | 5 (24) | − |
| Nasopharyngitis | 5 (24) | − |

^a^No grade 4 or 5 treatment-related AEs were reported.
AE, adverse event; ALT, alanine aminotransferase; AST, aspartate aminotransferase.

**Supplementary Table 3.** Adverse Events of Special Interest Related to Atezolizumab Occurring in ≥2 Patients or in Any Patient at Grade ≥3 (Safety-Evaluable Population)

| **Preferred term, n (%)** | **Any Grade** | **Grade 3^a^** |
| --- | --- | --- |
| **Any** | **18 (86)** | **9 (43)** |
| Rash | 12 (57) | 4 (19) |
| AST increased | 6 (29) | − |
| Blood bilirubin increased | 6 (29) | 2 (10) |
| ALT increased | 5 (24) | 2 (10) |
| Liver function test increased^b^ | 2 (10) | 1 (5) |
| Meningitis aseptic | 1 (5) | 1 (5) |
| Pneumonitis | 1 (5) | 1 (5) |

^a^No grade 4 or 5 events were reported.

^b^Term used by investigators if multiple liver enzyme tests were elevated in a single patient.

AST, aspartate aminotransferase; ALT, alanine aminotransferase.

**Supplementary Table 4.** Safety Summary (Safety-Evaluable Population)

| **Patients, n (%)** | **All (N = 21)** |
| --- | --- |
| Any AE | 21 (100) |
| Any treatment-related AE | 20 (95) |
| Grade 3–5 AE | 14 (67) |
| Any treatment-related Grade 3–5 AE | 12 (57) |
| Grade 5 AE | 0 |
| Serious AE | 8 (38) |
| AE leading to atezolizumab withdrawal^a^ | 7 (33) |
| AE leading to alectinib withdrawal^b^ | 4 (19) |
| AE leading to dose modification/interruption | 16 (76) |

AE, adverse event.

^a^Liver enzymes increased (n = 2; onset Day 44, related to both study treatments, and onset Day 20, related to atezolizumab only); pain and pyrexia (n = 1, same patient); neutrophil count decreased (n = 1, onset Day 178, related to alectinib only), nausea (n = 1), pneumonitis (n = 1), and rash (n = 1).

^b^Liver function test increased (n = 2), pain and pyrexia (n = 1, same patient), and neutrophil count decreased (n = 1).

**SUPPLEMENTARY FIGURES**

**Supplementary Figure 1.** Treatment duration and confirmed investigator-assessed response (RECIST v1.1; efficacy-evaluable population).

**
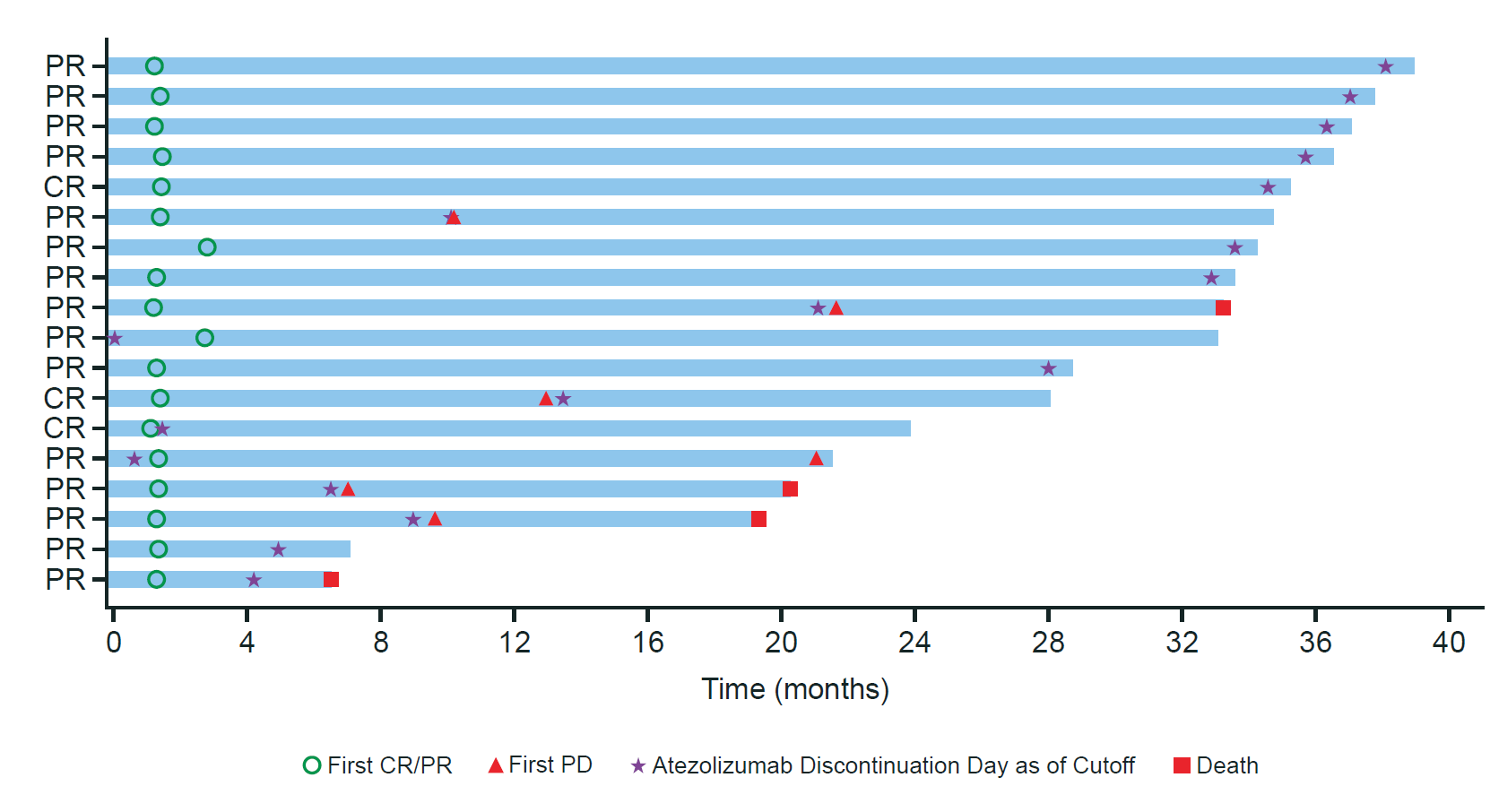
**

CR, complete response; PD, progressive disease; PR, partial response; RECIST, Response Evaluation Criteria in Solid Tumors.

**Supplementary Figure 2.** Tumor burden over time by confirmed investigator response (efficacy-evaluable population). **
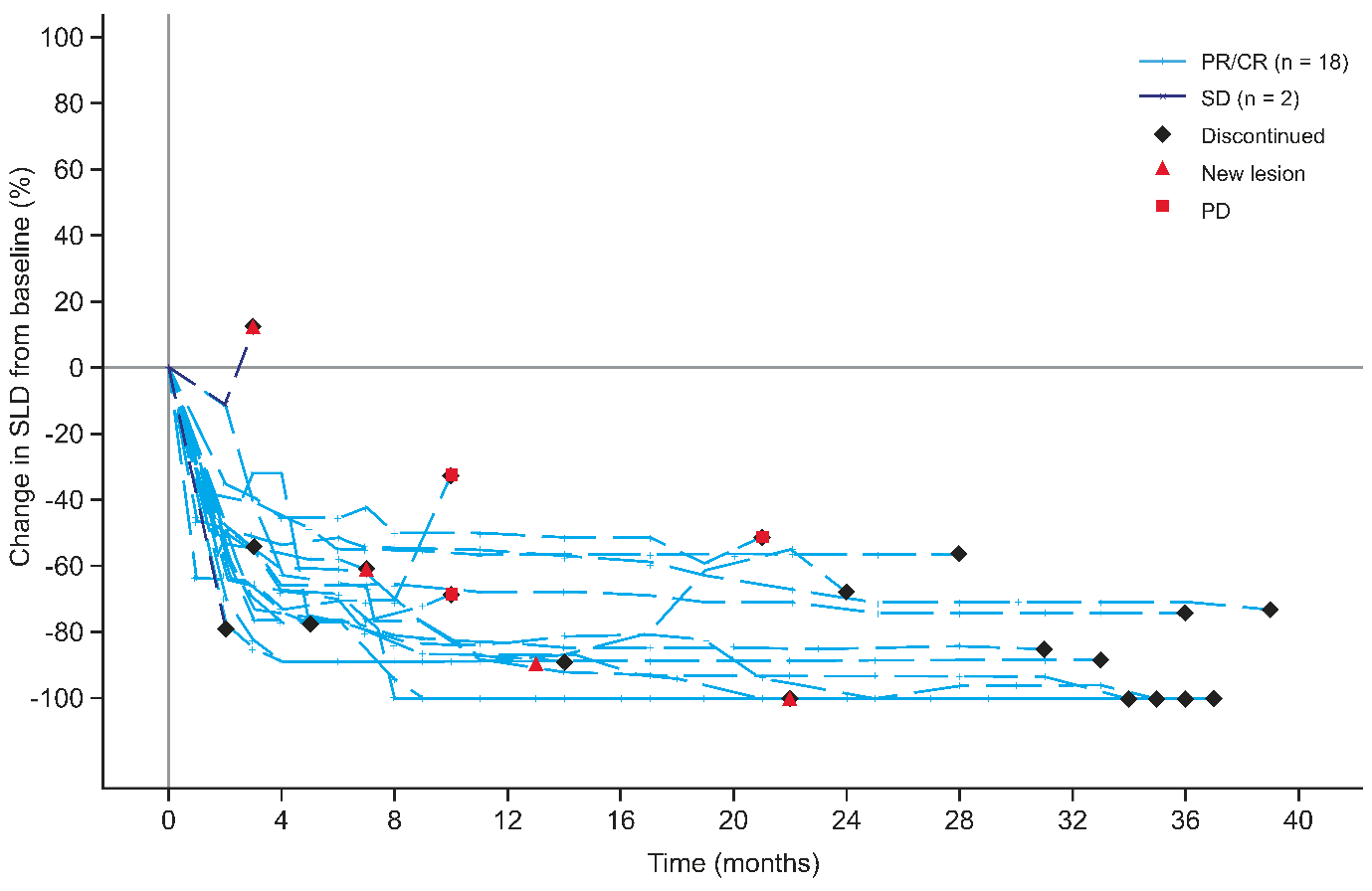
**

CI, confidence interval; CR, complete response; NE, not estimable;
PD, progressive disease; PR, partial response; SD, stable disease; SLD, sum of longest diameter.**Supplementary Figure 3.** Kaplan-Meier analysis of time to event outcomes (*A*) progression-free survival and (*B*) overall survival (efficacy-evaluable population).**
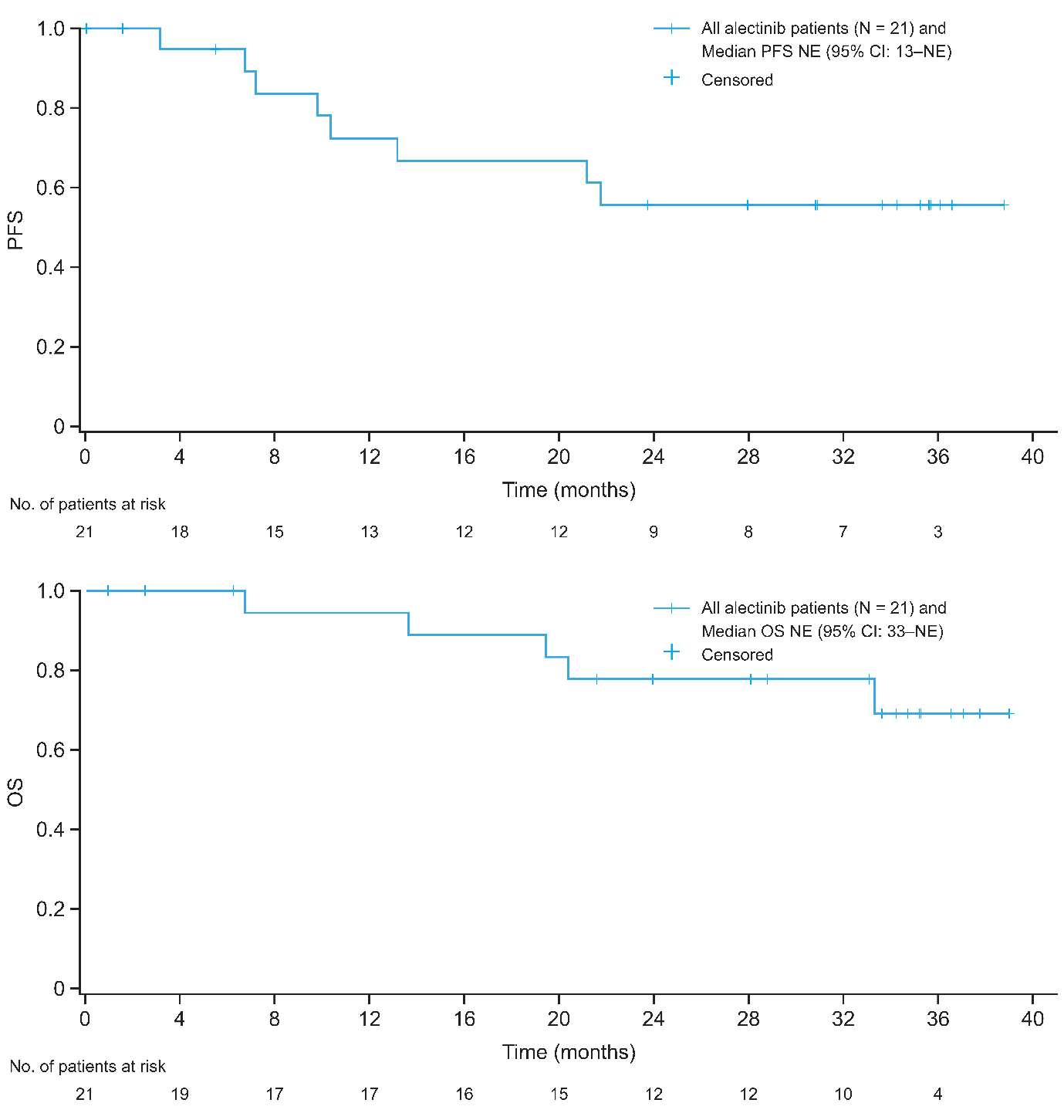
**

CI, confidence interval; NE, not estimable; OS, overall survival; PFS; progression-free survival.

**Supplementary Figure 4.** Patient baseline biomarker status.

**
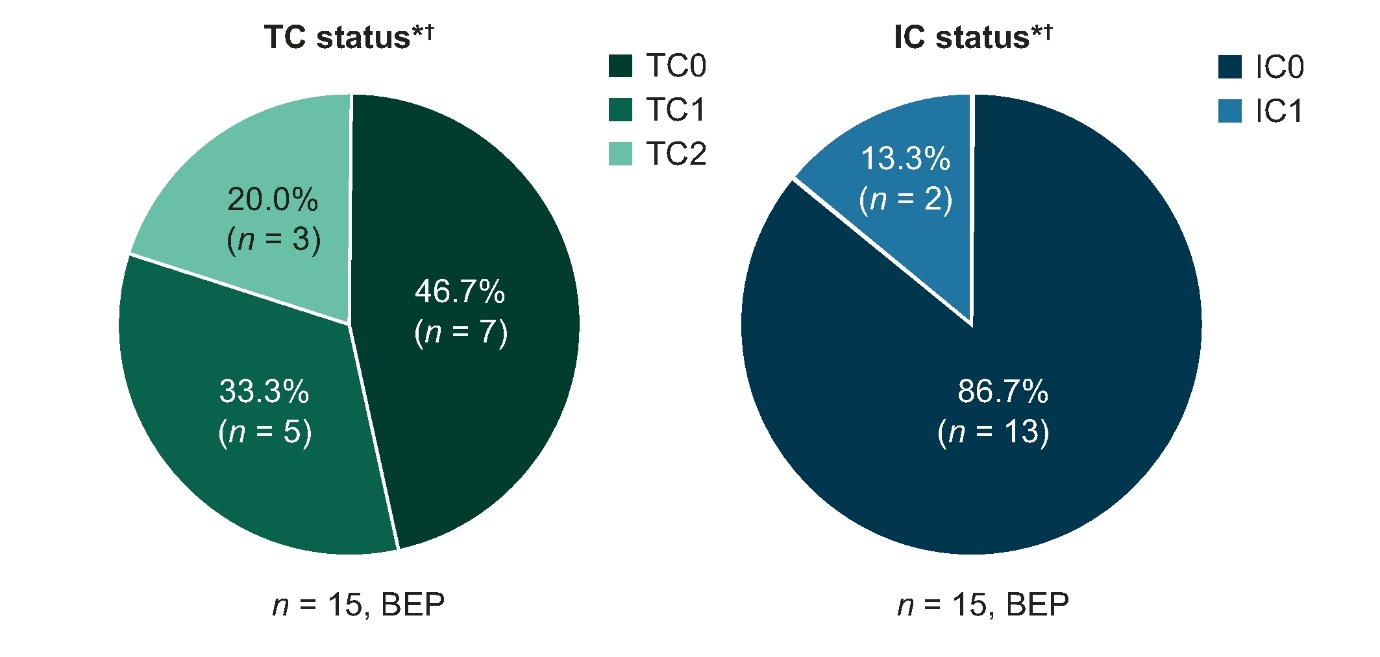
**

*PD-L1-positive cells defined as: TC0 and IC0 (<1%), TC1 or IC1 (≥1% but <5%); TC2 (≥5% but <50%).

^†^One patient had a pre-treatment sample taken >2 years before C1D1; all others had pre-treatment samples taken within 1 month of C1D1.

Abbreviations: BEP, biomarker-evaluable population; C1D1, Cycle 1 Day 1; CD8, cluster of differentiation 8; IC, tumor-infiltrating immune cell; PD-L1, programmed death-ligand 1; TC, tumor cell.

**Supplementary Figure 5.** Percentage change in CD8+ T cells observed pre- and post-treatment with alectinib from biopsies taken on Day 7 of Cycle 1.

**
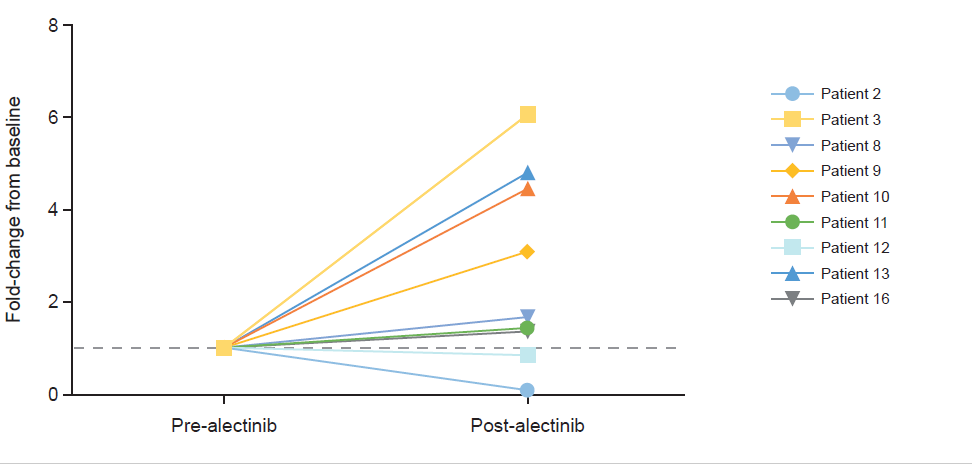
**

Abbreviations: CD8, cluster of differentiation 8.
